# Considerations for transition from subcutaneous to oral prophylaxis in the treatment of hereditary angioedema

**DOI:** 10.1186/s13223-021-00603-9

**Published:** 2021-10-09

**Authors:** Richard G. Gower, Mary Wilber

**Affiliations:** Marycliff Clinical Research, 820 S McClellan St #414, Spokane, WA 99204 USA

**Keywords:** Hereditary angioedema, Treatment burden, Quality of life, Prophylactic treatment, Shared decision-making, Berotralstat, Lanadelumab, Switching treatment

## Abstract

**Background:**

Hereditary angioedema (HAE) is a rare genetic disorder characterized by unpredictable localized episodes of edema, which is frequently managed with long-term prophylactic medications. Until recently, long-term prophylaxis has predominantly required regular intravenous or subcutaneous administration, however the recent approval of berotralstat (Orladeyo™) offers an orally administered prophylactic which may be associated with a lower burden of treatment compared to injectable options for some patients.

**Case presentation:**

This report describes four participants in the APeX-S trial who transitioned from subcutaneously administered lanadelumab (Takhzyro^®^) to daily oral berotralstat for long-term HAE prophylaxis. Lanadelumab dosing continued after berotralstat commencement in all patients and was tapered before discontinuation in three of the four patients. No substantial increases in HAE attack rates were observed after the transition to berotralstat monotherapy. One patient experienced a treatment-related adverse event (dyspepsia), which was mild and self-resolving.

**Conclusions:**

All four patients described in this case series successfully transitioned from lanadelumab to berotralstat monotherapy for long-term prophylaxis without significant complications and without the use of a complex transition protocol. The decision to transition to berotralstat monotherapy and how the transition should be achieved was discussed between patient and physician, ensuring that the comfort and perspectives of the patients were considered during the treatment transition. This report highlights the importance of individualization of HAE management plans to address both the disease and treatment burdens of HAE, and thus to provide the best possible quality of life for each patient.

## Background

Hereditary angioedema (HAE) is a rare genetic disorder commonly caused by deficient (type 1) or dysfunctional (type 2) C1-esterase inhibitor (C1-INH) protein [1], a serine protease inhibitor that regulates the kallikrein-bradykinin cascade [2]. A deficiency of functional C1-INH leads to increased bradykinin production, causing increased vascular permeability and angioedema [2]. The overall prevalence of HAE is approximately 1:50,000, and the incidence is similar among males and females [3, 4]. More than 95% of HAE cases are caused by mutations in serine protease inhibitor G1 (SERPING1), which codes for C1-INH [2]. Approximately 450 different mutations in SERPING1 have been reported [2].

HAE symptoms manifest as unpredictable, recurrent, localized episodes of edema in the extremities, face, trunk, genitalia, and gastrointestinal and upper respiratory tracts [1, 2]. Although many episodes occur unpredictably, some attacks may be triggered by environmental stressors including physical trauma, emotional distress, and infection [5]. The gastrointestinal tract is a common site of angioedema, which can cause severe pain and potentially lead to bowel obstruction and unnecessary surgical interventions [1, 6]. Moreover, patients with HAE have an underlying risk of mortality because of the possibility of upper airway occlusion caused by laryngeal angioedema [1, 7, 8]. Due in part to the unpredictability of attacks and the chronic nature of the disease, HAE can have profound adverse effects on health-related quality of life, including the domains of work, education, and mental health [9, 10]. In a 2017 survey of 445 patients with HAE, 50% of respondents reported mild to severe anxiety and 24% reported depression [11].

Currently, no curative treatment exists for HAE. Therefore, many patients choose to manage their symptoms with long-term administration of prophylactic medications that reduce the frequency and severity of attacks [3]. In addition, short-term prophylaxis can be administered immediately before the known attack triggers occur, and on-demand treatment can be used at the onset of an attack to reduce the severity and duration of angioedema [1, 3, 12]. Historically, the available treatment options for HAE prophylaxis were limited to attenuated androgens such as danazol, antifibrinolytics such as tranexamic acid, and intravenously (IV) administered plasma-derived C1-INH (IV-C1-INH) replacement therapies such as Cinryze^®^ (Takeda, Lexington, MA) [3, 12].

Since 2017, newer options for long-term HAE prophylaxis with better efficacy, safety, and routes of administration have become available, such as the subcutaneously (SC) administered C1-INH (SC-C1-INH) concentrate Haegarda^®^ (CSL Behring, King of Prussia, PA), which offers greater convenience and improved maintenance of steady-state plasma C1-INH concentration than does IV-C1-INH (Cinryze) [3, 12, 13]. Furthermore, lanadelumab (Takhzyro; Takeda, Lexington, MA), an SC administered fully human IgG1 monoclonal antibody that selectively inhibits plasma kallikrein, was approved by the FDA in 2018 with a recommended injection schedule of every 2 weeks, whereas SC-C1-INH requires twice-weekly administration [14, 15]. IV-C1-INH (Cinryze), SC-C1-INH, and lanadelumab are all recommended as first-line long-term HAE prophylaxis by the medical advisory board of the US Hereditary Angioedema Association [3].

Subcutaneous options for long-term HAE prophylaxis are associated with a reduced treatment burden and increased ease of self-administration in comparison to IV prophylactics [1, 16]. However, even though the SC route is perceived as less invasive than the IV route, SC injections are associated with injection-site reactions, including pain, and may cause anxiety in patients who are uncomfortable with self-injection [17–21]. Furthermore, effective self-administration of SC therapies typically requires patient education and training (necessitating adequate time and resources), and the injection process itself can be time-consuming [22, 23].

Berotralstat (Orladeyo; BioCryst, Durham, NC) is a highly selective orally administered plasma kallikrein inhibitor, available for long-term HAE prophylaxis in patients aged 12 years and older [24]. In the double-blind, placebo-controlled, phase 3 APeX-2 clinical trial, the rate of HAE attacks from baseline through 24 weeks of treatment was reduced significantly for both the 110 mg and 150 mg daily doses of berotralstat relative to placebo [25]. The rate for placebo recipients declined from 2.91 (baseline) to 2.35 (week 24) attacks per month, whereas berotralstat 110 mg daily reduced the attack rate from 2.97 to 1.65 attacks/month (*P* = 0.024 vs. placebo) and the 150-mg dose reduced the attack rate from 3.06 to 1.31 attacks/month (*P* < 0.001 vs. placebo) [25]. The most common treatment-emergent adverse events (≥ 10% in any of the three treatment arms) were upper respiratory tract infection, nausea, abdominal pain, vomiting, diarrhea, headache and back pain [25]. Long-term prophylaxis with oral berotralstat is being investigated further in an open-label study in patients with type 1 or type 2 HAE: APeX-S (NCT03472040). The primary endpoint of the study is safety and the secondary endpoints are efficacy, quality of life, and treatment satisfaction [26].

Because berotralstat is given orally, it may be associated with a lower treatment burden compared to injectable prophylactics such as IV-C1-INH (Cinryze), SC-C1-INH, and lanadelumab for some patients. In a 2020 survey of 75 patients with HAE, 86% of respondents reported that although they were satisfied with their current prophylaxis, they would still be interested in a medication that is easier to administer, and 61% expressed a preference for receiving treatment more discreetly [27].

To improve patient quality of life, it is important to reduce the treatment burden of HAE prophylaxis and to provide treatment compatible with patients’ lifestyles, preferences, and experiences. A safe and effective oral prophylactic may be preferred to the current standard of care by some patients, particularly those who are averse to a long-term injection regimen. However, the optimal approach for switching a patient to oral berotralstat prophylaxis has not been defined. Factors to consider include the timing of the transition, the half-life of the medications, the dosing schedules for both drugs, and patient-specific characteristics that may affect the transition.

Herein we report on four participants in the APeX-S trial (NCT03472040) who switched from biweekly SC lanadelumab 300 mg to daily oral berotralstat 150 mg for long-term HAE prophylaxis. Treatment decisions were individualized to each patient and made in partnership between the patient and HAE physician in each case. This report is intended to provide health care professionals with examples of safe transitions from lanadelumab to oral berotralstat prophylaxis. All reported information represents interim data from the APeX-S trial.

## Case presentations

### Case 1

This case describes a 24-year-old male with a family history of HAE, who was diagnosed with type 1 HAE when he was 1 month old, and initially presented with HAE symptoms at 8 years of age. He was enrolled in the APeX-S study in 2019. Prior to enrollment in the study, the patient self-administered icatibant (Firazyr^®^; Takeda) for on-demand treatment of HAE attacks; he also received IV-C1-INH (Berinert^®^; CSL Behring) in the emergency department as needed. He had previously received IV-C1-INH (Cinryze) as long-term HAE prophylaxis, followed by biweekly lanadelumab for approximately 3 years prior to study entry. In the 6 months prior to the screening phase of the APeX-S study, he experienced approximately one HAE attack per month on lanadelumab monotherapy.

The patient experienced mild dyspepsia shortly after commencing berotralstat treatment, which was deemed related to berotralstat by the investigator. Within 4 weeks, the dyspepsia had resolved spontaneously without any treatment or lifestyle change.

**Fig. 1:**
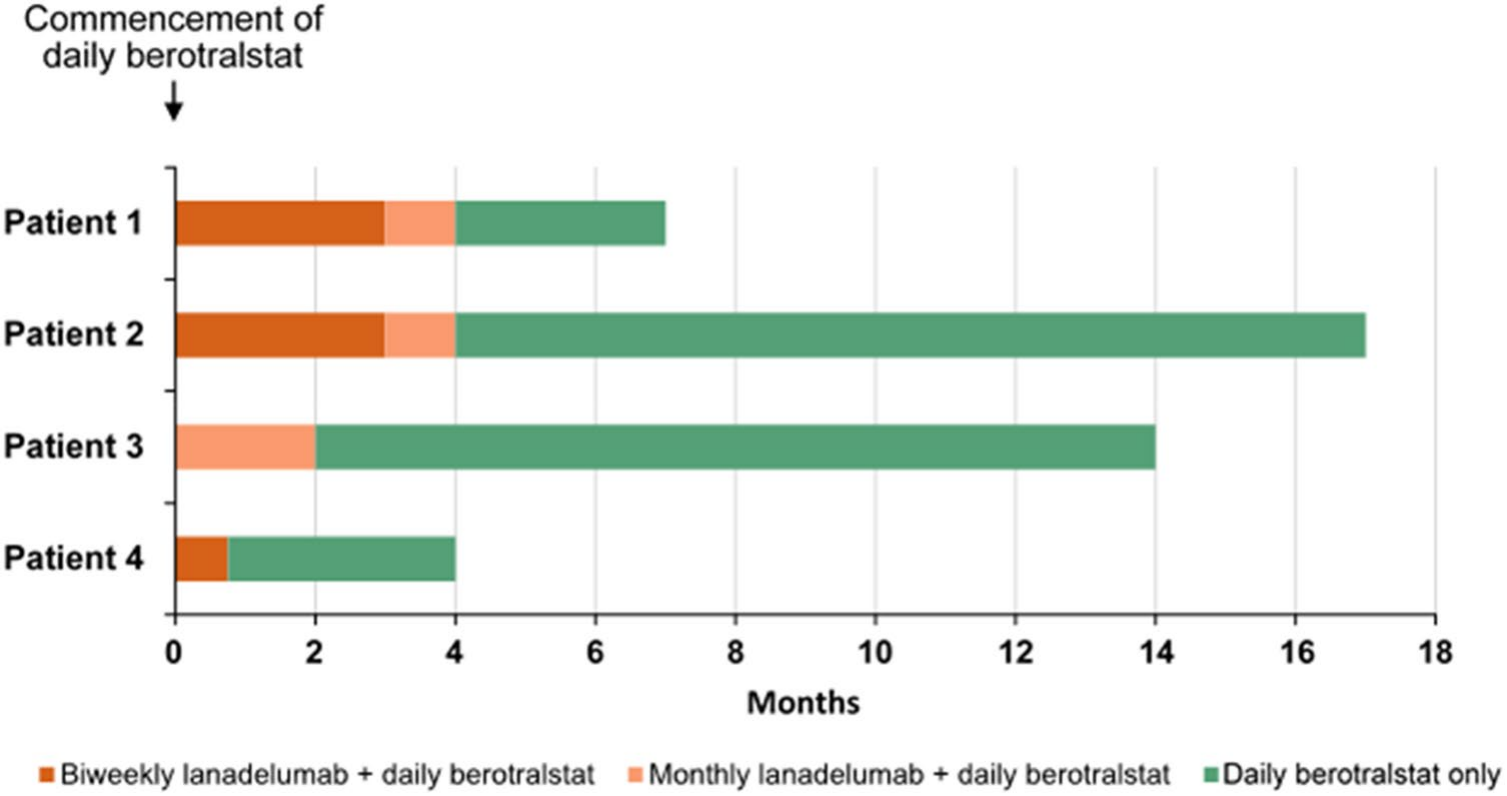
Medication doses during trial period

For the first 4 months of daily berotralstat treatment, the patient continued biweekly lanadelumab dosing; its discontinuation was delayed due to scheduled dental surgery. He experienced three HAE attacks during this dual-treatment phase, one of which was treated with icatibant and medications for pain and nausea; the two other attacks did not require treatment. When dental surgery was indefinitely postponed, the patient was transitioned off lanadelumab by reducing treatment dosing to every 4 weeks for the final month (Fig. 1).

The patient experienced two HAE attacks in the subsequent 3 months of berotralstat monotherapy, which represented a lower monthly rate of HAE attacks compared to the 6 months of lanadelumab monotherapy prior to study entry. Neither of these attacks required on-demand HAE treatment, but one was treated with medications to relieve pain and nausea. The patient experienced some work-related logistical challenges in taking oral berotralstat with his largest meal each day, which led to a decrease in adherence after 24 weeks of berotralstat treatment. Berotralstat therapy was eventually discontinued for personal reasons, and the patient is no longer enrolled in the study.

### Case 2

This case describes a 47-year-old female with a family history of HAE, who was diagnosed with type 1 HAE at 19 years of age, and for whom the symptoms of HAE first emerged at 12 years of age. In 2019, the patient enrolled in the APeX-S trial. She had previously received IV-C1-INH (Cinryze) for long-term HAE prophylaxis, but then switched to biweekly lanadelumab, which she received for approximately 3.5 years before enrollment in the study. After transitioning to lanadelumab, she continued using IV-C1-INH (Cinryze) and/or icatibant for on-demand treatment of HAE attacks. On average, the patient experienced one HAE attack per month on lanadelumab prophylaxis in the 6 months prior to screening.

Upon initiation of daily berotralstat, lanadelumab administration was continued for approximately 4 months: dosing was biweekly for the first three months, then every 4 weeks for one month (Fig. 1). During this 4-month period of dual therapy, the patient experienced two HAE attacks, both of which required treatment with icatibant and IV-C1-INH (Cinryze).

She subsequently received berotralstat monotherapy for 13 months, during which she experienced 4 HAE attacks: one was treated with both icatibant and IV-C1-INH (Cinryze), and two were treated with icatibant alone. Hence, the HAE attack rate was lower with berotralstat monotherapy compared with lanadelumab prophylaxis in the 6 months before screening. No treatment-related adverse events were observed during the time she received berotralstat. The patient maintained a high level of adherence to berotralstat throughout, and her self-reported treatment satisfaction compared favorably with baseline at most time points.

### Case 3

This case describes a 16-year-old female with a family history of HAE, who was diagnosed with type 1 HAE at 6 years of age, following initial onset of symptoms at age 5. She was enrolled in the APeX-S study in 2019, at which time she had been prescribed icatibant and IV-C1-INH (Cinryze) by her treating provider for on-demand treatment of HAE attacks. She had previously received IV-C1-INH (Cinryze) as long-term prophylaxis, before switching to bi-weekly lanadelumab for roughly 2.5 years before enrollment in the study. She had not experienced an HAE attack in the preceding 18 months on lanadelumab prophylaxis.

When daily berotralstat treatment began, lanadelumab dosing was switched to a monthly regimen for 2 months before it was discontinued (Fig. 1). No HAE attacks occurred in this dual-therapy phase. In the subsequent 12 months of berotralstat monotherapy, the patient experienced two HAE attacks, one of which required on-demand treatment with icatibant. The severity of the two attacks was self-assessed as moderate (abdominal discomfort, headache, and erythema marginatum) and negligible, respectively.

During the first 2 months of berotralstat treatment, the patient had some difficulty with adherence. After re-education by the treating physician, mean adherence improved to more than 90%. The patient reported excellent satisfaction with berotralstat treatment and experienced no treatment-related adverse events.

### Case 4

This case describes a 25-year-old female with a family history of HAE, who was diagnosed with type 1 HAE when she was 4 years old and for whom the onset of HAE symptoms occurred at 12 years of age. She had previously received IV-C1-INH (Cinryze) for long-term prophylaxis, before switching to biweekly lanadelumab monotherapy, which she received for 15 months prior to her enrollment in the APeX-S study in 2020. In the 6 months leading up to study enrollment, the patient experienced an average of 2 HAE attacks per month with lanadelumab prophylaxis.

After starting oral berotralstat, the patient continued self-administration of biweekly lanadelumab for one month before it was discontinued (Fig. 1). The patient experienced two HAE attacks during this 4-week dual therapy phase, both requiring icatibant treatment. In the first 2.5 months of berotralstat monotherapy, the patient did not experience any HAE attacks, but 3 attacks occurred in the subsequent 2 weeks, two of which were self-assessed as mild and the other as moderate in severity. Two of these attacks were triggered by physical activity, and no identifiable trigger was reported for the third. All three attacks required icatibant treatment. Regardless, the patient’s attack rate during the 3 months of treatment with berotralstat monotherapy was lower than during the 6 months of lanadelumab monotherapy prior to study entry. No treatment-related adverse events were identified while she received berotralstat. Her level of satisfaction with this treatment was excellent at all time points; however, she is no longer enrolled in the study for personal reasons.

## Discussion

This report describes the successful transition of four patients with type 1 HAE from lanadelumab to berotralstat monotherapy for long-term prophylaxis. The transition was straightforward and largely uneventful in all cases. Decisions relating to each transition plan were made as a partnership between the physician and the patient; key considerations included patient preferences, quality of life, and level of comfort with the new medication, in addition to safety and disease control. Given that long-term prophylaxis may be needed for many years, a change in medication is likely to be of great significance to patients’ perception of attack risk. Therefore, it is important for each patient to feel comfortable and to be able to participate in clinical decision-making concerning their disease.

A dual-therapy phase, as allowed by the study protocol, was conducted with all four cases in this report to ease patient anxiety and provide time for the patient to become comfortable with berotralstat. In three of the four patients, lanadelumab dosing was tapered during the dual-therapy phase. It takes 6 to 12 days from initiation of daily berotralstat to achieve steady-state concentration [24], and the terminal elimination half-life of lanadelumab is approximately 2 weeks [14]. Therefore, neither tapering nor treatment overlap are medically necessary to maintain a sufficient level of HAE prophylaxis if daily berotralstat is commenced concurrently with the final dose of lanadelumab, and an extended overlap period may not be a realistic option in real-world clinical practice. A comparison of a dual-therapy phased transition with an immediate switch from lanadelumab to berotralstat may provide useful insights into this aspect of the transition.

Another important consideration in the timing of each transition is to ensure it does not coincide with any known attack triggers. In this case series, all four patients reported that their attacks, although broadly sporadic and unpredictable, could be triggered by factors including physical injury (4 patients), physical activity (4 patients), stress and anxiety (3 patients), infections or colds (3 patients), and dental or other medical procedures (3 patients). For example, the patient described in case 1 was scheduled to undergo dental surgery near the time of transition and had previously required intensive care (including intubation) after surgery triggered an HAE attack despite preoperative administration of IV-C1-INH. Lanadelumab discontinuation was delayed intentionally to ensure the patient was comfortable with his level of protection against HAE attacks.

Gastrointestinal adverse effects, such as that experienced by the patient in case 1, are the most common adverse events associated with berotralstat; they typically occur soon after treatment initiation, then reduce in frequency and self-resolve with time [24, 25]. It is recommended that berotralstat is taken with a meal to minimize gastrointestinal effects [24], and that adverse events and drug reactions are recorded in a patient diary that should be reviewed as part of regular follow-up [3].

The level of convenience and treatment burden of any HAE prophylactic, and thus the ability of patients to sustain sufficient adherence to treatment, will vary according to patient-specific factors such as lifestyle and level of comfort with their HAE treatment – factors that may change over time. For example, the patient described in case 1 was sometimes unable to take berotralstat with a large meal, making it difficult for him to maintain adherence. In addition, the adolescent patient in case 3 initially did not adhere sufficiently to berotralstat treatment; however, after re-education by her physician on the importance of HAE prophylaxis, her adherence increased to more than 90% and remained high thereafter. HAE management should be periodically reviewed and discussed with the patient [3]. Although findings of studies involving other diseases differ on the optimal route and frequency of administration for maximal treatment adherence [28–30], a qualitative review of 102 articles demonstrated that convenience of administration is likely to have a favorable effect on adherence [31].

Berotralstat adherence exceeded 90% for all patients in this case series; however, it should be noted that these patients received berotralstat as part of a clinical trial, and adherence is likely to be lower in real-world settings. Some elements of the transition strategy described herein, such as a prolonged dual-therapy phase, are also unlikely to be approved for reimbursement in real-world clinical settings. The key limitation of this study is its preliminary nature: there were a small number of cases, and each followed a different transition schedule. This prevents definite conclusions from being drawn. More research is needed to define clear recommendations for safe and effective transition from lanadelumab to oral berotralstat, including the optimal timing of this transition, that can be applied in real-world practice.

## Conclusions

Berotralstat is a safe and effective oral option for long-term HAE prophylaxis, with the potential to reduce the burden of treatment compared with the current standard of care. The successful transition of four patients from SC lanadelumab to berotralstat for long-term HAE prophylaxis reported here supports the feasibility of this transition in clinical practice and provides a preliminary model of how it may be conducted. The transition was uneventful and relatively simple in all patients, and though a dual-therapy phase was employed in this study, immediate transition to berotralstat monotherapy may be possible in many instances. These cases emphasize the importance of shared decision-making between the patient and physician to ensure that the patient’s comfort and concerns are fully considered. HAE management plans should be individualized to each patient’s disease characteristics, perspectives, and lifestyle so that patient quality of life is prioritized, and the burdens of disease and treatment are minimized.

## Data Availability

The datasets generated and/or analyzed during the current study are not publicly available due to being interim data from a clinical trial but are available from BioCryst on reasonable request.

## Abbreviations

C1-INH: C1-esterase inhibitor
FDA: Food and drug administration
HAE: Hereditary angioedema
IV: Intravenous
SC: Subcutaneous
SERPING1: Serine protease inhibitor G1
US: United States
